# A Comparison Between the Effects of Single-Dose Oral Gabapentin and Oral Clonidine on Hemodynamic Parameters in Laparoscopic Surgeries: A Randomized Controlled Trial

**DOI:** 10.7759/cureus.37251

**Published:** 2023-04-07

**Authors:** L Gayathri, Anand Kuppusamy, Gunaseelan Mirunalini, Karthik Mani

**Affiliations:** 1 Anesthesiology, Meenakshi Hospital, Thanjavur, IND; 2 Anesthesiology, SRM Medical College Hospital and Research Centre - SRMIST, Chennai, IND

**Keywords:** endotracheal intubation, gabapentin, clonidine, laparoscopy, hemodynamics

## Abstract

Background and objective

Laparoscopic surgeries can result in exaggerated sympathetic responses due to pneumoperitoneum. Many drugs including clonidine and gabapentin have been evaluated to attenuate the hemodynamic response to abdominal insufflation. In light of this, this study was conducted to compare the effects of preoperative administration of oral gabapentin with those of clonidine on intraoperative hemodynamic parameters in patients undergoing laparoscopic surgeries.

Methodology

A prospective, randomized, double-blinded comparative trial spanning a period of one year was conducted involving 150 patients scheduled to undergo elective laparoscopic surgeries at a tertiary hospital. Patients who fulfilled the inclusion criteria were randomly allocated into three groups: to receive oral gabapentin 300 mg (Group G) or oral clonidine 150 mcg (Group CL) or a placebo tablet (Group C). Standard anesthetic protocols were followed during the surgery and the mean arterial pressure (MAP), heart rate (HR), postoperative pain as assessed by visual analog scale (VAS) scores, postoperative analgesic consumption, sedation scores, and complications like nausea, vomiting, and dry mouth were recorded and analyzed.

Results

HR and MAP were significantly reduced in the intervention groups (clonidine and gabapentin) compared to the control group. There was a statistically significant reduction in MAP and HR in patients on oral clonidine compared to patients on gabapentin. Postoperative pain as assessed by VAS score was better in the intervention groups compared to patients who were administered a placebo. Postoperative analgesic consumption was significantly lower in patients on clonidine and gabapentin compared to the control group. Patients on oral gabapentin received lower doses of tramadol compared to patients on clonidine. Postoperative sedation as assessed by the Ramsay sedation scale (RSS) score was higher in patients on oral gabapentin. Complications like postoperative nausea and vomiting were significantly reduced in the intervention groups, while dryness of mouth was more prevalent in patients on clonidine.

Conclusion

Based on our findings, oral clonidine is more effective in attenuating hemodynamic response to pneumoperitoneum compared to oral gabapentin. Postoperative pain was lower in intervention groups compared to the control group. However, patients on gabapentin required a lower dose of analgesics postoperatively compared to patients on clonidine. Postoperative sedation was also more pronounced in patients on gabapentin while dryness of mouth was more common in patients on oral clonidine.

## Introduction

Laparoscopic surgeries are minimally invasive, modern surgical techniques; they entail small incisions and are associated with minimal postoperative pain, better cosmetic results, shorter recovery time, early enteral feeds, less intraoperative bleeding, and fewer postoperative respiratory complications [1]. Despite all these advantages, laparoscopic surgeries have certain demerits due to physiological changes during pneumoperitoneum and positioning. Hence, anesthetic techniques for laparoscopic surgery must be refined, as it can often be challenging for the anesthesiologist [2].

Pneumoperitoneum created during laparoscopy results in hypercapnia, which in turn causes adverse cardiac effects such as tachycardia, hypertension, an increase in systemic vascular resistance, and a decrease in cardiac output [3]. The adverse cardiac effects due to pneumoperitoneum can be aggravated by the pressor response to laryngoscopy and endotracheal intubation [4,5]. Many pharmacological agents like clonidine and gabapentin have been evaluated as oral premedications; moreover, intravenous drugs such as vasodilators (nitroglycerine), opioids (fentanyl), beta-blockers (esmolol), and calcium channel blockers can be administered prior to abdominal insufflation to attenuate the hemodynamic response to pneumoperitoneum [6,7]. This stress response can also be obtunded by deepening the plane of anesthesia by the use of volatile anesthetic agents or by administering large doses of opioids like fentanyl (5-10 µg/kg). Though pain after laparoscopic surgeries is less intense, a multimodal approach by combining nonopioid drugs and regional anesthesia with opioids to decrease the dose-related side effects of opioids is usually preferred [8].

Gabapentin, a structural analog of gamma-aminobutyric acid (GABA), is used as an anti-epileptic agent and is effective in suppressing neuropathic pain. The drug acts by binding to the α2δ subunit (heterodimeric GABA-B receptors) of the presynaptic voltage-gated calcium channels and inhibits calcium release. It also acts on the N-methyl-D-aspartate (NMDA) receptor, which inhibits the substance P and glutamate [9]. Gabapentin has been used as an adjuvant in the management of acute postsurgical pain and to decrease postoperative opioid requirements [10]. The mechanism by which gabapentin blunts the pressor response to laryngoscopy and pneumoperitoneum is unclear. Clonidine is a selective alpha-2 agonist that specifically stimulates the α2A subtype. These receptors are present in both presynaptic and postsynaptic autonomic ganglia in the central and peripheral nervous systems. This results in the reduction of sympathetic outflow, which attenuates the hemodynamic response to any surgical nociceptive stimulus.

Our analysis of the literature revealed few studies comparing clonidine with gabapentin during laparoscopic surgeries. Hence, we conducted this study with the primary objective to compare between effects of oral clonidine and oral gabapentin as well as a placebo in attenuating hemodynamic response during abdominal insufflation in laparoscopic surgery. The secondary objectives were to compare postoperative pain, analgesic consumption, postoperative sedation, and complications.

## Materials and methods

A prospective double-blinded randomized control trial was conducted at a tertiary hospital in south India over a period of one year (2019-2020). After obtaining Institutional Ethics Committee (1772/IEC/2019) approval and Clinical Trial Registry of India (CTRI/2020/03/023971) registration, 150 patients were randomly allocated into three groups by computer-generated random numbering. Patients with American Society of Anesthesiologists (ASA) 1 and 2 physical status, aged 18-65 years, weighing 40-80 kg, and undergoing surgery for a duration of up to two hours were included in this study. Patients with uncontrolled systemic illness, a history of drug abuse, and an allergy to study drugs, those who refused to enroll, those in whom difficult airways were anticipated, and those on sedatives, hypnotics, and analgesics for chronic pain were excluded from the study. Ethical principles such as respect for the patient, beneficence, and justice were strictly adhered to and the study was conducted as per the tenets of the Declaration of Helsinki.

All patients were assessed for anesthesia on the day before surgery and were kept fasting according to hospital protocol. Patients in the gabapentin group (Group G), those in the oral clonidine group (Group CL), and those in the control group (Group C) received oral gabapentin 300 mg, oral clonidine 150 mcg, and placebo tablet respectively two hours before surgery with sips of water. General anesthesia was induced with Inj. fentanyl 2 mcg/kg, Inj. propofol 2mg/kg, and the trachea was intubated with Inj. vecuronium 0.1 mg/kg. Anesthesia was maintained with sevoflurane 1-2% and O_2_/air mixture with a fraction of 50% inspired O_2_ and Inj. vecuronium was supplemented as per neuromuscular monitoring. The flow rate of gas to create pneumoperitoneum was kept at 3 liters per minute and intra-abdominal pressure was maintained between 13 and 15 mmHg throughout the surgery for all patients. Pulse rate, mean arterial pressure (MAP), and SpO_2 _were monitored continuously for all patients by a dedicated anesthesiologist. The data for heart rate (HR) and MAP were collected from the time of peritoneal insufflation and thereafter every 10 minutes till the end of surgery. After recovery from anesthesia, patients were shifted to the post-anesthetic care unit (PACU) for monitoring and observation. The time taken to attain a modified Aldrete score of 9 and above was recorded as the recovery time. Postoperative pain was assessed using the visual analog scale (VAS) every four hours till 24 hours. Inj. paracetamol 1 g IV was given if the VAS score was >4. If pain persisted after 30 minutes of paracetamol infusion, Inj. tramadol 100 mg was given intravenously. Total tramadol and paracetamol consumption was noted. Sedation score was assessed every four hours till 24 hours as per the modified Ramsay sedation scale (RSS), ranging from 1-6 as follows: 1: anxious, agitated, restless; 2: cooperative, oriented, tranquil; 3: responds to commands only; 4: brisk response to a light glabellar tap or loud noise; 5: sluggish response to a light glabellar tap or loud noise; 6: no response.

Sample size estimation

The sample size was estimated based on changes in HR in three groups as the primary variable with G*Power software version 3.1.9.4 by using the analysis of variance (ANOVA) test. This study was designed as a non-inferiority trial and, to detect a difference of 10%, we needed at least 47 patients per group. The power of the study was 80%. Hence, we included 50 patients in each group with a total of 150 study participants. All the statistical analysis was performed using IBM SPSS Statistics version 21 (IBM Corp., Armonk, NY). After checking for the normality of the data, one-way ANOVA was used to test continuous variables, and the Chi-square test was used to test the categorical variables. Post hoc analysis was performed using Tukey’s honest significance test for pair-wise comparison of means. Frequencies were expressed as percentages and ordinal data were tested using the Kruskall-Wallis test. A two-sided p-value <0.05 was considered statistically significant.

## Results

**Figure 1:**
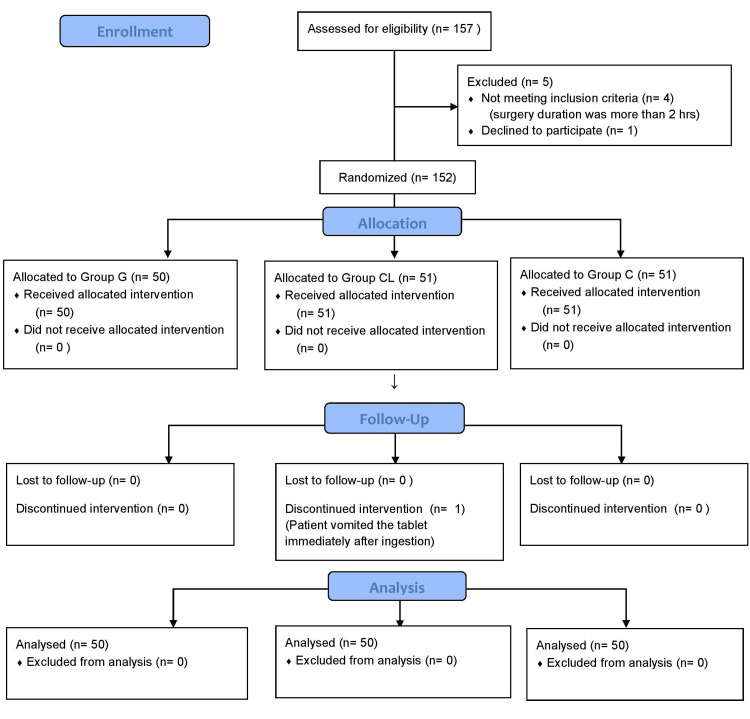
CONSORT diagram CONSORT: Consolidated Standards of Reporting Trials

The Consolidated Standards of Reporting Trials (CONSORT) flow diagram illustrating the inclusion of participants in the study is depicted in Figure 1.

There was no statistically significant difference between groups CL, C, and G in terms of demographic characteristics of the patients such as age, sex, ASA status, body weight, and duration and type of surgery (Table 1).

**Table 1:** Demographic characteristics *Not significant ASA: American Society of Anaesthesiologists; SD: standard deviation

Variables		Group CL	Group G	Group C	P-value
Age, years, mean ±SD		37.3 ±9.8	36.7 ±8.8	37.1 ±9.9	0.94^*^
Sex, M/F		24/26	21/29	29/21	0.27^*^
ASA, I/II		24/26	22/28	25/25	0.82^*^
Weight, kg, mean ±SD		62.5 ±10.1	62.1 ±9.6	62.3 ±10.1	0.97^*^
Duration of surgery, minutes, mean ±SD		106.9 ±11.2	104.6 ±7.4	105.5 ±11.1	0.50^*^
Type of surgery	Laparoscopic cholecystectomy	16	14	15	0.98^*^
	Laparoscopic appendicectomy	18	18	17	
	Laparoscopic meshplasty	16	18	18	

We observed that MAP was significantly lower in patients premedicated with oral clonidine and gabapentin compared to patients on a placebo up to 12 hours postoperatively. Intergroup comparison between clonidine and gabapentin revealed a significant reduction in MAP in patients administered clonidine from 40 minutes to two hours postoperatively, after which the values were comparable (Table 2).

**Table 2:** Comparison of mean arterial pressure ^Ϯ^Significant SD: standard deviation

Time	Intraoperative mean arterial pressure			P-value	
	Group CL, mean ±SD	Group G, mean ±SD	Group C, mean ±SD	P-value for comparison of 3 groups	P-value for comparison between Groups CL and G
Baseline	89 ±6.80	91 ±10.74	90 ±10.37	0.47	0.2057
10 mins	78 ±8.18	79 ±12.55	85 ±9.64	0.003^Ϯ^	0.6753
20 mins	78 ±7.63	81 ±11.79	86 ±10.61	0.001^Ϯ^	0.106
30 mins	76 ±8.46	79 ±11.72	89 ±12.15	<0.001^Ϯ^	0.2413
40 mins	75 ±8.95	77 ±12.84	91 ±13.27	<0.001^Ϯ^	0.4867
50 mins	76 ±7.66	77 ±9.95	91 ±13.12	<0.001^Ϯ^	0.0001^Ϯ^
60 mins	72 ±7.64	78 ±9.49	92 ±12.62	<0.001^Ϯ^	0.0014^Ϯ^
70 mins	74 ±6.67	75 ±7.97	91 ±11.92	<0.001^Ϯ^	0.0001^Ϯ^
80 mins	74 ±7.20	76 ±8.83	91 ±10.94	<0.001^Ϯ^	0.0001^Ϯ^
90 mins	74 ±5.92	76 ±6.99	90 ±10.09	<0.001^Ϯ^	0.0001^Ϯ^
100 mins	73 ±6.30	77 ±7.70	90 ±9.56	<0.001^Ϯ^	0.0001^Ϯ^
110 mins	76 ±6.50	79 ±8.74	91 ±9.07	<0.001^Ϯ^	0.0001^Ϯ^
120 mins	74 ±5.69	79 ±7.26	91 ±8.29	<0.001^Ϯ^	0.0001^Ϯ^

The mean HR was better controlled in Group CL and Group G when compared to Group C before induction, after induction, after intubation, and throughout the procedure. The association between the control group (Group C) and intervention groups (Groups CL and G) with respect to HR was statistically significant (p<0.0001). Intergroup comparison (Group CL and Group G) of HR showed better control in the clonidine group and the difference was statistically significant (p<0.001). The distribution of HR is shown in Table 3.

**Table 3:** Comparison of heart rate ϮSignificant SD: standard deviation

Time	Heart rate			P-value	
	Group CL, mean ±SD	Group G, mean ±SD	Group C, mean ±SD	P-value for comparison of 3 groups	P-value for comparison between Groups CL and G
Baseline	78.12 ±6.693	79.84 ±12.046	78.4 ±8.398	0.613	0.379
10 mins	67.24 ±7.367	76.9 ±12.888	91.68 ±8.265	<0.001^Ϯ^	<0.001^Ϯ^
20 mins	67.2 ±7.812	75.18 ±12.603	92.24 ±8.248	<0.001^Ϯ^	<0.001^Ϯ^
30 mins	66.34 ±8.29	75.46 ±12.104	92.76 ±8.13	<0.001^Ϯ^	<0.001^Ϯ^
40 mins	67.04 ±7.827	74.4 ±11.436	92.12 ±7.311	<0.001^Ϯ^	<0.001^Ϯ^
50 mins	67.78 ±7.416	74.78 ±10.088	90.84 ±7.517	<0.001^Ϯ^	<0.001^Ϯ^
60 mins	68.32 ±7.391	74.2 ±10.144	89.36 ±7.661	<0.001^Ϯ^	<0.001^Ϯ^
75 mins	68.78 ±6.95	74.62 ±9.651	88.16 ±7.372	<0.001^Ϯ^	<0.001^Ϯ^
90 mins	69.16 ±6.619	74.38 ±9.019	87 ±7.332	<0.001^Ϯ^	<0.001^Ϯ^
105 mins	69.76 ±6.173	74.7 ±8.765	85.32 ±7.133	<0.001^Ϯ^	<0.001^Ϯ^
120 mins	70.56 ±6.148	75.02 ±8.445	83.72 ±7.137	<0.001^Ϯ^	0.003^Ϯ^

The mean postoperative analgesic consumption was also significantly reduced in Group CL and Group G when compared to Group C. There was no significant difference in postoperative analgesic consumption between patients on clonidine and those on gabapentin (Table 4).

**Table 4:** Comparison of postoperative analgesic consumption ^Ϯ^Significant SD: standard deviation

Postoperative analgesics	Group CL, mean ±SD	Group G, mean ±SD	Group C, mean ±SD	P-value for comparison of 3 groups	P-value for comparison between Groups CL and G
Paracetamol dose in grams	0.86 ±0.85	0.82 ±0.83	2.2 ±0.4	<0.001^Ϯ^	0.811
Tramadol dose in milligrams	82 ±0.66	58 ±0.61	168 ±10.2	<0.001^Ϯ^	0.002^Ϯ^

**Figure 2:**
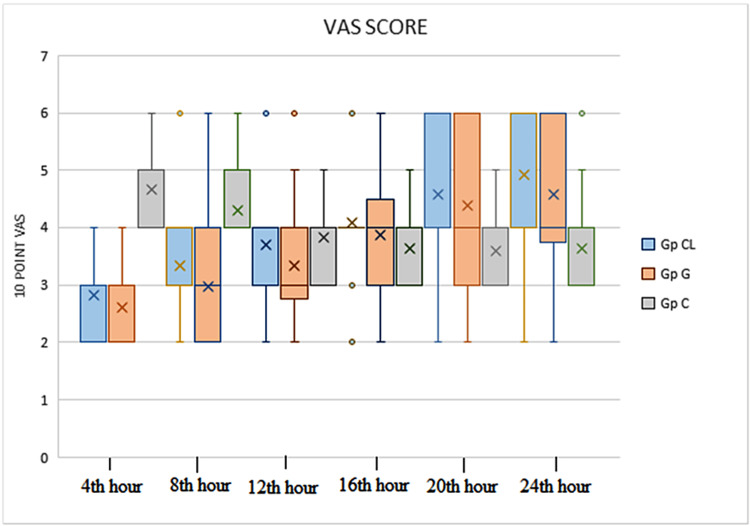
Box plot of postoperative visual analog scale score VAS: visual analog scale

Postoperative pain as assessed by mean VAS score was significantly reduced in Group CL and Group G up to 20 hours when compared to Group C, but there was no statistically significant difference in VAS score for pain between Group G and Group CL (Figure 2).

**Figure 3:**
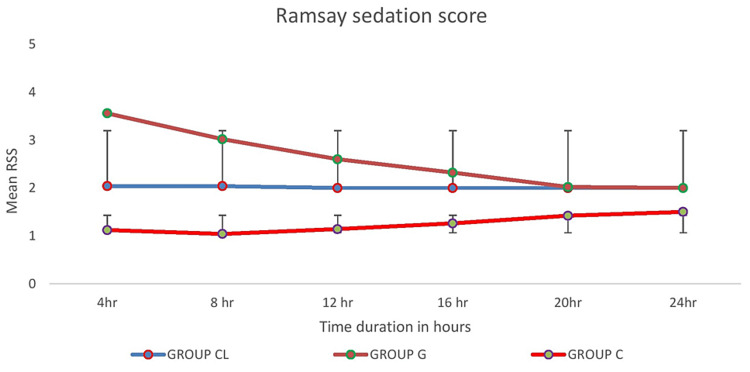
Comparison of postoperative sedation based on Ramsay sedation scale score RSS: Ramsay sedation scale

Postoperative sedation as assessed by RSS score was significantly higher in Group CL and Group G than in the control group up to 24 hours. The intergroup comparison revealed statistically significant postoperative sedation in Group G for up to 16 hours when compared to Group CL (Figure 3).

**Figure 4:**
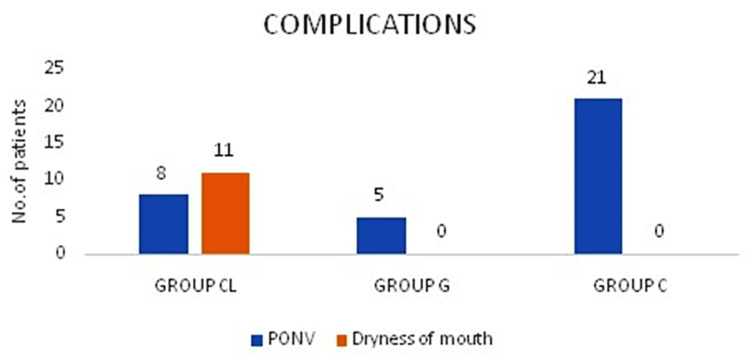
Postoperative complications PONV: postoperative nausea and vomiting

Postoperative nausea and vomiting were less prevalent in Group CL (16%) and Group G (10%) when compared to Group C (42%). Dryness of mouth was more prevalent in Group CL (Figure 4).

## Discussion

In our study, patients in all three groups were comparable in terms of various demographic parameters like age, sex, ASA status, weight, and type and duration of surgery. Our study demonstrated a statistically significant decrease in MAP in Group CL and Group G when compared to patients in the control group throughout the procedure. The MAP was significantly lower in Group CL after abdominal insufflation, compared to Group G, probably due to the synergistic, α2 action of clonidine and hypotension due to the induction agent. Our results are comparable to those of Kapse et al. [10] who compared oral clonidine (5 mcg/kg) and oral gabapentin 800 mg given 90 minutes prior to surgery, which showed a significant difference in MAP. Also, our results are in concordance with Singhal et al. [5] who compared oral clonidine 200 mcg and gabapentin 900 mg administered 90 minutes before induction, which showed blood pressure below baseline in the clonidine group in comparison with the gabapentin group. However, our results are slightly different from those of the study conducted by Majumdar et al. [4] in which oral clonidine (200 mcg) and oral gabapentin (600 mg) were administered before surgery, and clonidine was found to be better at attenuating hemodynamic parameters before induction, during laryngoscopy, and orotracheal intubation at zero, first, third, and fifth minutes while gabapentin attenuated HR and MAP at seventh and 10th minutes. This may be due to the high dose of gabapentin administered in this trial compared to our study.

In our study, patients in Group CL and Group G had a significant reduction in HR during pneumoperitoneum compared to patients in Group C. This significant reduction in HR is comparable to the findings of Kapse et al. [5], Singhal et al. [10], and Waikar et al. [11], which showed a statistically significant reduction in HR in clonidine and gabapentin groups during endotracheal intubation and carbon dioxide insufflation. Clonidine, as an alpha-2 adrenergic agonist in the nucleus tractus solitarius (NTS), excites a pathway that inhibits excitatory cardiovascular neurons. Clonidine has an effect on the posterior hypothalamus and ventromedial rostral-ventrolateral areas of the medulla, resulting in a decreased sympathetic outflow from the central nervous system (CNS), which causes a reduction in blood pressure and HR.

Postoperative pain was significantly decreased up to 20 hours and postoperative consumption of analgesics was reduced in Group CL and Group G in comparison to the control group. Patients in the gabapentin group received a lower dosage of tramadol when compared to the clonidine group, thus demonstrating gabapentin has better analgesic efficacy compared to clonidine. This is probably due to the action of gabapentin on multiple pain pathways like NMDA and voltage-sensitive calcium channels. This is in line with the study done by Hassani et al. [9] comparing oral gabapentin (1000 mg) and placebo, which showed a statistically significant increase in the duration of postoperative analgesia in patients on gabapentin than placebo groups. Similar results were obtained in a clinical trial by Pandey et al. [12] where the postoperative analgesia requirement was significantly lower in the gabapentin group compared to the tramadol and control groups. The superior analgesic profile of gabapentin was confirmed in a clinical trial done by Rupniewska-Ladyko et al. [13] wherein the time to administer rescue analgesics was prolonged in the gabapentin group compared to the control group. Our results are similar to those of a clinical trial by Alayed et al. [14] in which preoperative gabapentin led to a significant reduction in the consumption of morphine at 24 hours. Gabapentin produces significant analgesia due to its action on glutamate neurotransmission and on voltage-sensitive calcium channels.

In our study, postoperative sedation was statistically higher in patients in the clonidine and gabapentin groups up to 24 hours when compared to the control group. On comparing patients in Group G with those in Group CL, sedation was statistically higher in Group G up to 16 hours compared to the CL group. Our results correlate with those of the study done by Kapse et al. [10] in which patients had better sedation in the gabapentin group, which is due to the antagonism of NMDA receptors.

The incidence of postoperative nausea and vomiting was 16% in the clonidine group, 10% in the gabapentin group, and 42% in the control group. Dryness of mouth was more prevalent in the clonidine group (22%) when compared to the gabapentin group due to its alpha-2 agonistic action. Similar results were obtained in a study conducted by Alayed et al. [14] in which nausea was less prevalent in the gabapentin group than in the control group. Patients who needed an antiemetic drug were significantly fewer in the gabapentin group when compared to the placebo group in a study done by Ajori et al. [15].

Limitations

This study has a few limitations. Since all laparoscopic procedures in our study lasted less than two hours, we could not compare hemodynamic parameters over a longer time frame. We also did not use patient-controlled analgesia postoperatively since most of the procedures were daycare surgeries, in which opioid usage is restricted.

## Conclusions

Based on our findings, the administration of oral clonidine and gabapentin before surgery is effective in maintaining hemodynamics during pneumoperitoneum compared to a placebo in laparoscopic surgeries. Post hoc analysis revealed that patients on oral clonidine had better control of hemodynamics than gabapentin while there was no significant difference between the two groups with respect to postoperative pain and analgesic requirement. Postoperative sedation level was higher in patients on oral gabapentin compared to those on clonidine.
